# Giant brain tuberculoma mimicking a malignant tumor in a child

**DOI:** 10.11604/pamj.2014.17.112.3941

**Published:** 2014-02-14

**Authors:** Ali Akhaddar, Mohamed Boucetta

**Affiliations:** 1 Department of Neurosurgery, Avicenne Military Hospital, Marrakech, Morocco; 2 University of Mohammed V Souissi, Rabat, Morocco

**Keywords:** Brain tumour, malignant tumor, tuberculoma, tuberculosis

## Image in medicine

A 6-year-old boy, with no past medical history, presented with progressive headache, vomiting, right side weakness since 3 months without fever or seizures. On examination, he was conscious with a stiff neck and right hemiplegia. Brain computed tomography-scan showed a heterogeneous calcified large mass-like lesion in the left fronto-temporo-parietal region, irregular contrast enhancement and extensive perilesional edema with important mass effect (A-C). There was a strong suspicion of malignant tumor. Routine blood tests and chest X-ray were normal. Magnetic resonance imaging was requested but not made because of the rapid deterioration of the state of consciousness of the child. The patient was operated urgently. At operation there was a large intra-axial yellowish, firm, and relatively avascular lesion. Total excision of the mass was done (D). Histological features were consistent with tuberculoma. Serological test for HIV was negative. The patient improved progressively and was discharged on antituberculous treatment with a good outcome. Intracranial tuberculoma should always be considered in the differential diagnosis of solitary and large focal brain lesions, particularly in patients of tuberculosis endemic areas. In our patient, surgical excision not only helped to establish the histological diagnosis but also helped to resolve the compressive symptoms.

**Figure 1:**
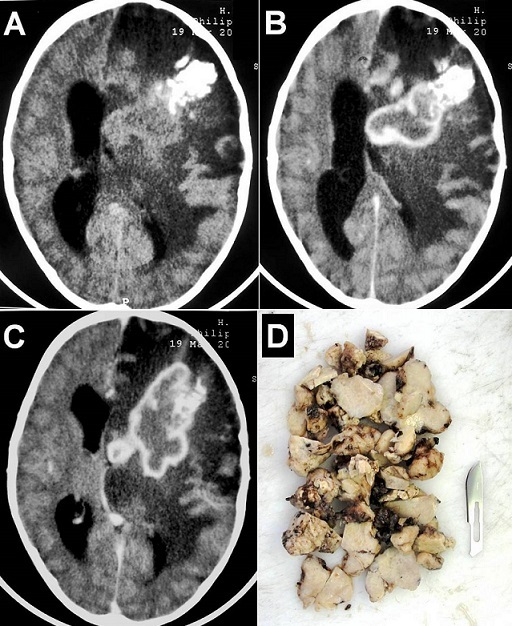
Brain computed tomography-scan before (A) and after contrast injection (B and C) showing a heterogeneous calcified large mass-like lesion in the left fronto-temporo-parietal region, irregular contrast enhancement and extensive perilesional edema with important mass effect. (D) External photograph of the large yellowish, firm, and relatively avascular tumor-like mass

